# Remarks on the Climate and Epidemic Diseases of Rangoon, Burman Empire

**Published:** 1852-05

**Authors:** John Dawson


*Remarks on the Climate and Epidemic Diseases of Rangoon, Burman Empire.* By John Dawson, M. D. Communicated by J. Remington, M. D., of Philadelphia.

In the provinces of Burmah bordering on the sea coast, the seasons are uniformly regular in their appearance, and steady in their continuance. The year is divided into two seasons, viz. the dry and wet. From May to October is the south-west, or wet monsoon; from November to April is the north-east, or dry monsoon. In the rains, it sometimes pours down literally in *torrents* and for hours together. The average fall of rain from year to year, for a period of ten years, is about 175 inches, the minimum being 160, and the maximum 502 inches. The first half of the dry season is commonly called here "the cool season," which corresponds in some respects to the winter of temperate latitudes. The next half is hot, *hot*, *hot*, week in and week out, and no rain. The whole face of the country becomes parched and dry. The very leaves of the trees wither from the effects of a scorching sun, and the birds pant and droop their heads for want of a cool shade. After the first few showers of rain have fallen in May, all nature wakes up to new life and begins to revive. At the end of that month and early in June, the country is once more clothed in luxuriant verdure. But from an extreme of dryness and heat, we find ourselves very speedily led into an extreme of dampness and wet. Every thing feels damp and becomes mouldy. Books go to pieces in two or three years. Furniture held together by glue, or other adhesive matter, opens at the joints. At the coldest season of the year, the thermometer is down to 60°. In the hottest it is up to 112°. These are the lowest and highest points in which the mercury ranges in this climate. Rangoon lies in latitude 62° 47′ North, and longitude 97° 15′ East of London.

Of epidemic diseases, there are but two which seem to visit Burmah, at least so far as I am now competent to judge, i. e., cholera, and small pox. There does not, however, appear to be any definite regularity in the time of their visitations. Except in the Tenasserim and Akracan Provinces, which are under the jurisdiction of the British Government, vaccination is entirely unknown. Inoculation is occasionally practised, though the manner of doing it, and the extent to which it is carried, I am at present unable to say. As the Burmese practitioners, however, appear to care little for the public welfare, and the government still less, there cannot be much done in this way to protect the people, or to modify the nature of that scourge. In the whole kingdom of Burmah, there is no hospital, no public dispensary, no almshouse, no asylum in existence, to which the suffering can look for aid, the "Missionary Dispensary" excepted. No provision is made by any one, either official or private, for meeting the wants of the poor, the afflicted, and the miserable. To be afflicted with leprosy, (and there are many such cases,) is "*a crime*," which exposes the sufferer to fine, imprisonment and slavery. To be insane is a *sin*, a *curse*, for which you receive but little pity or commiseration. The person so deranged, is supposed to be under the dominion of "*nats*," or evil spirits, for misdeeds committed in a former state of being ; and he is allowed to drag out a wretched existence as a species of punishment. Such is the amount of their sympathy, and such is the form of their benevolence for some of the worst afflictions to which our fallen humanity is subjected.

Of the prevailing classes of disease, there is nothing that is specially interesting to mention. Fevers are more commonly met with in the dry season, and abdominal complaints in the wet. Excepting a few cases of asthma and catarrh, pulmonary affections are rather rare. Diseases of the liver and spleen are more frequent. One case of well defined enlargement of the pancreas has come under my observation. The symptoms seemed to be very analogous to those of chronic dyspepsia, and obstructions in the transverse portion of the colon. The organ could be felt distinctly throughout the entire length. It had an indurated feeling, and pressure communicated soreness to the patient. I imagined that it was about four times its ordinary size. There were also evidences of an accumulation of fluid in the abdominal cavity. As the man came to see me but twice, taking on each occasion some laxative pills with him, the result of the case is not known. He lives at a distance in the jungle. An exceedingly painful disease of the stomach, which I have regarded as "*Gastralgia*," is very common among the inhabitants of this country. In a large number of cases, it baffles all the efforts of the Burmese practitioners to remove it, and when once it becomes chronic, it eventuates in a lingering train of dyspeptic symptoms with general emaciation and debility. The primary cause of it, is doubtless attributable to the kind of condiments which they use with their food. These are mostly sour fruits and sour vegetables, rendered pungent and stimulating by the addition of capsicum, onions, garlic, &c., which provoke acidity, and lead to local irritation in the submucous tissue. Moreover the Burmese are a nation of "*smokers.*" All smoke, from first to last, from oldest to youngest of them, and as soon as they are capable of being inducted into the fashion, children begin to chew betel—a compound, composed of slaked lime, catechu, tobacco, areca-nut, and the leaf of the betel vine, portions of each being put into the mouth at the same time, and all chewed together. It imparts a very beautiful vermilion tint (consequent on the reaction between the alkali and vegetable) to the mouth and lips. These habits of smoking and chewing in persons so young, conduce to deprive the stomach of a large quantity of saliva that is necessary to healthy digestion, and aided by their peculiar diet, must sooner or later develope the very trying gastralgic complaint called by the people "*Yeen-liat-na*," which might be looked upon, as a "*national* disease" among the Burmans. I have treated it as all my medical brethren would, and pretty generally with success, by antacids, laxatives, local depletion and counter-irritants, forbidding the use of any article that would be likely to interfere with the treatment, or prolong the affection.

In the surgical department the cases have been far more numerous, than those that are strictly medical, of those who have sought assistance at the dispensary. They are not only greater in numbers, but more various and interesting in character. To present you with a bird's eye view of this division of practice, I will just give the names of the most common diseases of this class, that come seeking help. The eye has the greatest variety, viz.: Granular lids; simple conjunctivitis; opacity of the cornea in its different forms of nebula, leucoma, oblonga and staphyloma both of the cornea and sclerotic membranes, spherical and conical in shape; ectropium, pterygium, iritis, ulceration of cornea, capsular and lenticular cataract, amaurosis, and two or three kinds of ophthalmia, &c. Of other parts, the register kept exhibits cancer, internal hernia, stricture of the urethra, calculi in bladder, ulcers of different grades, simple and compound fractures, luxatio, subluxatio, primary and consecutive syphilis, gonorrhœa, hydarthrus, otalgia, periostitis, necrosis, deafness, incised wounds, abscess, &c. Most of these cases are of a very difficult and complicated nature, and a majority of them are of many years standing. The Burmese doctors here in vain tried to relieve them; and as a last resource some of them have come hundreds of miles to see and consult the "white doctor from a foreign county." The confidence they manifest towards me is at once remarkable and encouraging. But there is one serious drawback in the management of their cases, and that is, the reluctance they invariably evince to the performance of operations. They have a terrible dread of the knife, and on no account will they submit to be cut, unless you pledge them success. As this cannot be done for various reasons, the opportunities for using the knife are very limited. In this country, too, justice is a mere symbol, for in the event of a prosecution, however unreasonable it may be in a civilized community, the object is to fleece the foreigner. As an instance of it, the following facts will show. A few days ago, two or three European gentlemen went out in the morning for a ride. Two of them reside here as merchants. The third was a stranger, belonging to the Bengal Pilot service, who left his station for the benefit of the voyage to sea. In proceeding slowly along the road, a Burmese woman ran up against his poney, (notwithstanding his efforts to get out of her way), and fell down. The rider instantly stopped and dismounted to ascertain whether the woman was hurt. As there appeared to be little the matter, he said at once, as she was poor, that he would give her a present of twenty rupees, about ten dollars. The woman seemed surprised and glad at her good fortune, and quite satisfied. Soon, however, a crowd gathered round, persuaded her to sit in a blanket, and that they would take her to the deputy governor to make her complaint, when she would get plenty of money out of the "*white foreigner.*" The case was heard. The English gentleman was floored. The deputy governor decreed that he should pay one hundred and ten rupees, or go into the stocks. He urged that a medical man should be requested to examine the extent of injury, if any had been received, and he would pay any amount. But no, the money must be paid, or he should be sent to jail, and placed in the stocks. To prevent his being so degraded, the gentleman paid the money. The woman got ten, the governor and his officers pocketed the remainder. Such is heathen justice. It speaks for itself. The maxim inculcated on us all in the Bible, "to be wise as a serpent and harmless as a dove," is not more necessary to be followed in any part of the wide world, than it is in this benighted land.

In regard to your inquiry, touching the existence of scarlatina in India, based on the assertion of Dr. Gregory, that it has never been seen, I am sorry that I should have to differ in my opinion from so distinguished an authority. But this is the more excusable from the fact that the Professor was never in the East, and had to depend wholly on the statement of others in the preparation of his work on the practice of medicine. A few cases given in detail would be, of course, conclusive evidence of its being noticed, but not having these to offer at present, I am constrained to withhold all conjecture till it is my power to furnish them. If I mistake not, the disease has been met with in southern India. In Maulmein, the principal city of the British Provinces in Burmah, it has occurred in several instances, among the children of Missionaries at that station. Hereafter I shall feel awake to this subject, and advise you of any cases which may come under my observation.

Since opening the "*Dispensary*" at this station on the 1st of June last, I have kept, not a nominal, but a numerical list of the actual number of applicants daily for relief. The lists are written separately for each month; and for the five months which have elapsed, they show an aggregate of six thousand, one hundred and thirty-four persons, whose cases have received attention, and more or less medicine. Of this number, the same individual may have appeared several different times. This arrangement I dislike, because it is not a clear exponent of the real number of cases that have been under treatment. But in a country like Burmah it is unavoidable, where to write down men's names would excite suspicion that there was some ulterior political object in view. The notes of several interesting cases are before me, some anomalous, others characteristic and well marked, but I regret that I have not now the time to copy them for transmission.

